# Use of Hyperspectral/Multispectral Imaging in Gastroenterology. Shedding Some–Different–Light into the Dark

**DOI:** 10.3390/jcm8010036

**Published:** 2019-01-01

**Authors:** Samuel Ortega, Himar Fabelo, Dimitris K. Iakovidis, Anastasios Koulaouzidis, Gustavo M. Callico

**Affiliations:** 1 Institute for Applied Microelectronics (IUMA), University of Las Palmas de Gran Canaria (ULPGC), Las Palmas de Gran Canaria 35017, Spain; hfabelo@iuma.ulpgc.es (H.F.); gustavo@iuma.ulpgc.es (G.M.C.); 2 Dept. of Computer Science and Biomedical Informatics, University of Thessaly, 35131 Lamia, Greece; diakovidis@dib.uth.gr; 3 Endoscopy Unit, The Royal Infirmary of Edinburgh, Edinburgh EH16 4SA, UK; akoulaouzidis@hotmail.com

**Keywords:** hyperspectral imaging, multispectral imaging, clinical diagnosis, biomedical optical imaging, gastroenterology, medical diagnostic imaging

## Abstract

Hyperspectral/Multispectral imaging (HSI/MSI) technologies are able to sample from tens to hundreds of spectral channels within the electromagnetic spectrum, exceeding the capabilities of human vision. These spectral techniques are based on the principle that every material has a different response (reflection and absorption) to different wavelengths. Thereby, this technology facilitates the discrimination between different materials. HSI has demonstrated good discrimination capabilities for materials in fields, for instance, remote sensing, pollution monitoring, field surveillance, food quality, agriculture, astronomy, geological mapping, and currently, also in medicine. HSI technology allows tissue observation beyond the limitations of the human eye. Moreover, many researchers are using HSI as a new diagnosis tool to analyze optical properties of tissue. Recently, HSI has shown good performance in identifying human diseases in a non-invasive manner. In this paper, we show the potential use of these technologies in the medical domain, with emphasis in the current advances in gastroenterology. The main aim of this review is to provide an overview of contemporary concepts regarding HSI technology together with state-of-art systems and applications in gastroenterology. Finally, we discuss the current limitations and upcoming trends of HSI in gastroenterology.

## 1. Introduction

Hyperspectral/Multispectral (HS/MS) imaging (HSI/MSI), also known as Imaging Spectroscopy, is a technology capable of overcoming the imaging limitations of the human vision based in white light (WL). In fact, HSI combines the features provided by two technologies that have been, for decades now, used separately i.e., digital imaging and spectroscopy. Digital imaging allows recording of the morphological features of a given scene, extracting information of different objects in regards to shape and textures. Spectroscopy deals with the interaction between the electromagnetic (EM) radiation and matter. While the capabilities of the human vision are restricted to a certain region of the EM spectrum (EMS), the visible spectrum that spans from 400 to 700 nm, most common HS commercial systems expand this spectral range from 400 to 2500 nm. Although there are HS cameras able to cover the EM up to 12 microns, such systems are restricted to certain applications that are out of the scope of this manuscript. HSI provides information in regions of the EMS that the human eye cannot see, revealing therefore substance properties that are normally unavailable to human beings. Furthermore, while the human eye is only capable of distinguishing three different wavelengths associated with the opsins of the retina (Cianopsin sensitive to 430 nm -*blue light*-; Cloropsin sensitive to 530 nm -*green light*-; and, Eritropsin sensitive to 650 nm -*red light*-), HS cameras can capture the EMS in hundreds of different narrow wavelengths, largely increasing the resolution to over what humans can see. On the other hand, MSI is based on the same principle of HSI with the main difference being that MSI is generally characterized by a lower number of spectral channels [1].

**Figure 1:**
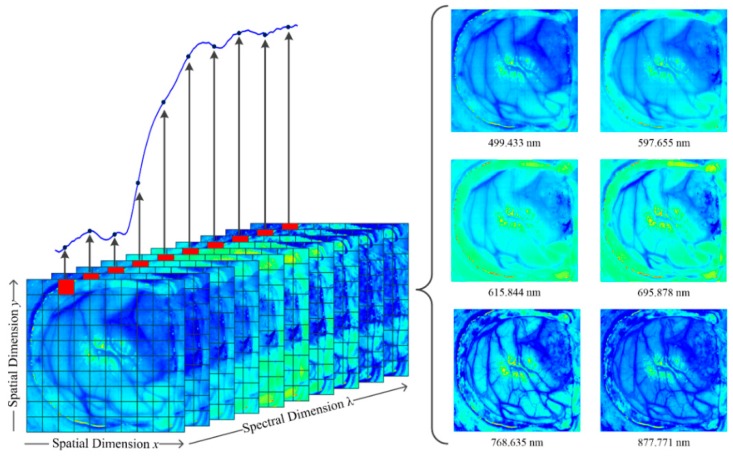
Example of an HS cube from in-vivo human brain surface and spectrum from the pixel in red (**left**). Several images at different wavelengths obtained from the HS datacube (**right**).

An HS image is recorded in a data structure called HS cube, which contains both spatial and spectral information from a given image. The information inside the HS cube can be visualized in several different ways. If a single pixel from an HS image is selected, the spectrum related to this pixel can be examined. Likewise, it is possible to visualize the entire spatial information for a given wavelength. The observed information at various wavelengths represents different properties of the matter. Figure 1 shows an HS cube, where both types of representations can be observed.

The spectral signature (also called spectral fingerprint) is the curve that links the EM radiation with a certain material. The key point of this concept is that each material has its own interaction with the EMS, hence the spectral signature of any given material is unique. By analyzing the spectral signatures contained in an HS image, it is possible to distinguish between the different substances that are present in the captured image. Nevertheless, to properly differentiate materials by using the spectral signature information, some issues have to be addressed. First, the measured spectral signature from the same material can present subtle variations, i.e., inter-sample variability. Second, there are materials that present spectral similarities among them, being extremely challenging to perform an automatic differentiation of such materials based only on their spectral signatures.

Many researchers have employed HSI technology for different applications [1] such as non-invasive food quality inspection [2,3], improving recycling processes [4], or examining paintings for accurate identification of the pigments used in order to refine their restoration [5,6]. Geologists use HSI to identify the location of different minerals [7]. Furthermore, in agriculture this technology has been used to quantitatively characterize the soil [8] or to identify the stress levels of plants [9].

**Figure 2:**
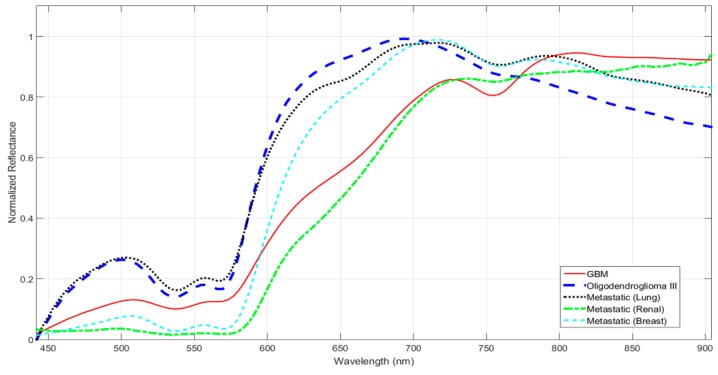
Spectral signatures of different brain tumor tissues in the VNIR (Visible and Near Infrared) range [10]. The abscissa axis represents the different wavelengths, and the ordinate axis represents the normalized reflectance. The continuous red line corresponds to Glioblastoma (GBM); the dashed blue line corresponds to Oligodendroglioma grade III; the dashed black, green and cyan lines correspond to a metastatic lung, renal and breast tumors, respectively.

Figure 2 presents an example of the spectral signatures of different tissues [10], where the differences between the spectral signatures of primary (glioblastoma and oligodendroglioma grade III) and secondary brain tumors (metastatic lung, renal and breast) are evident. Just a visual inspection of the shape of the reflectance curve reveals that it is possible to identify the type of tissue present at each pixel of the image. The measured spectral signatures are mainly affected by illumination, the mixture of different substances, and/or by noise.

In biomedical applications, the spectral signature is employed as an indicator of the different biochemical constituents of different tissues [11]. The spectral signature is a useful tool to differentiate among different tissues, and also to provide information useful to discriminate healthy from diseased tissue [12]. Nevertheless, spectral signatures of the same tissue from different subjects present differences due to biological variability. This fact is called inter-patient variability of data. Furthermore, the spectral signatures measures from different parts of the same tissue also present subtle differences. This is called intra-patient variability of data. Handling the intra- and inter-patient variability of data is one of the most important challenges in biomedical HS image analysis.

Due to the large amount of information that HSI provides, it is necessary to process and analyze the acquired HS images by using high-performance computational techniques, thus focusing on the information that is more useful for a particular application. In this review, we briefly discuss the different applications of HSI in the medical field and the HS systems and algorithms more commonly used as well as the current investigations performed in the application of HSI to study and diagnostic gastrointestinal (GI) diseases using in-vitro, ex-vivo and in-vivo samples.

## 2. Medical Hyperspectral Imaging

This section is intended to provide some context on the use of HS technology in the medical field prior to analyzing details of the use on HS in GI medicine. However, for a deeper introduction about the use of HSI in biomedical applications we strongly recommend the review articles published by Li et al. [13], Lu et al. [14] and Calin et al. [15]. Furthermore, for a better understating about light tissue interactions and how this information can be used in diagnostic applications, we recommend the studies performed in [11,12].

In the first decade of the 21st century, HSI has attracted the interest of researchers in the medical field for two main reasons. First, it has been proven that the interaction between the EM radiation and tissues carries quantitative diagnostic information about tissue pathology [11,12]; and second, because of the non-invasive nature of this technology. Recently, clinicians have started to use HSI in a number of situations. Some aim to detect cholesterol by analyzing HSI of the face [16] or arthritis by studying the skin reflectance [17]. HSI has also been used for detecting peripheral arterial disease [18], to enhance the visualization of blood vessels [19] or to achieve automatic differentiation between veins and arteries during surgery [20]. This technology has also been used to measure the oxygenation levels of retina [21], brain [22], or kidney [23].

In detection of neoplasia, the main aim of using HSI is to develop aid-visualization tools able to accurately delineate the boundaries of the tumor in order to improve resection of cancerous lesions, hence avoiding the unnecessary resection of healthy tissue. This technology has been successfully applied in detecting prostate cancer [24], head and neck cancer [25,26] or breast cancer [27] in animal models. In humans, this technology has been employed for the detection of tongue cancer [28], oral cancer [29], skin tumors [30,31,32] or brain cancer [10,33].

In the field of histopathology, where the current diagnostic techniques are based on the morphological analysis of tissue specimen slides, HSI can be employed as a complementary source of information that may unburden the workload of the pathologists. Researchers have proven the capabilities in detecting several diseases using this technology, such as examining retina sections for the quantitative assessment and evaluation of the effect of medication [34], detecting cancer metastasis in lung and lymph nodes tissue [35], identifying brain tumors [36] or lung cancer [37].

In the following sections, we briefly introduce the main concepts involved in medical HSI and the use of HSI as a new technology for assessing the detection of GI diseases.

## 3. Hyperspectral Systems

HS acquisition systems present a challenge to engineers, who have to handle sophisticated optical and electronic systems to generate an HS cube. There are several types of HS cameras, however depending on how data are acquired the main categorization is in spatial scanning cameras and spectral scanning cameras [38].

*Spatial scanning* cameras, based on the push-broom technique, are capable of acquiring simultaneously a single spatial dimension (a narrow line of an image) and the whole spectral information for a given scene. To capture an HS cube in this manner, it is necessary to perform a spatial scanning, where either the camera or the captured object(s) shift their position while the camera is capturing frames. The scanning can be also performed by using a mirror in front of the fore optic, and moving the mirror to image the whole object. Although the use of mirrors allows developing more compact instrumentation (hence more appealing in clinical circumstances), it is necessary to take care regarding the geometric distortions mirrors can produce in the captured image. The core of these cameras is an optical element that splits the incoming radiation into specific wavelengths values [39].

**Figure 3:**
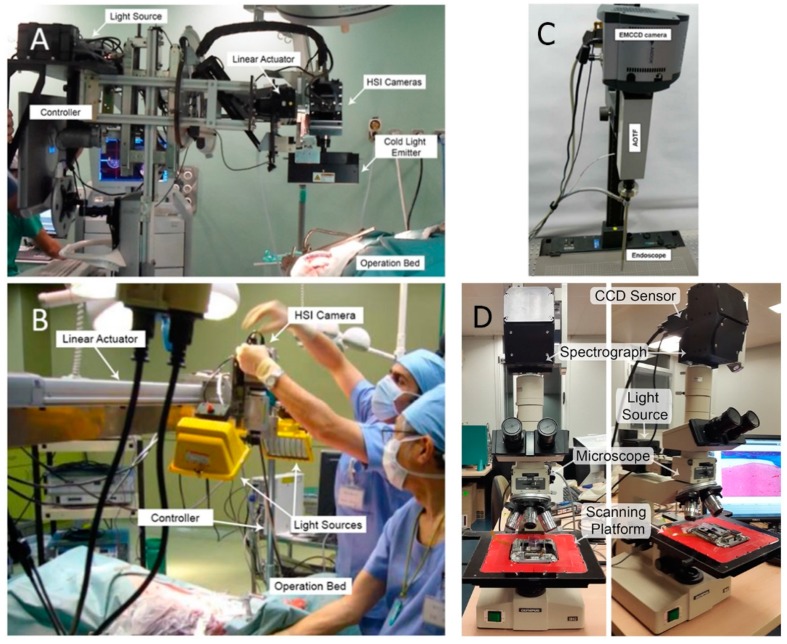
HS acquisition system used in medical applications. (**A**,**B**) HSI acquisition systems based on push-broom cameras for in-vivo human brain tumor detection [40] and in-vivo pig abdominal surgery [20]; (**C**) Liquid crystal tunable filter camera attached to an endoscope for cancerous tissue detection [43]; (**D**) Microscope coupled to an HSI push-broom camera for pathological slides registration [36].

This type of camera has the advantage of capturing images with high spectral resolution, offering also an excellent trade-off between spatial and spectral resolutions, compared to other HS cameras, in the expense of performing scanning in order to acquire an HS cube. For this reason, in the medical field these types of cameras are used in open surgical procedures, for in-vivo surface inspection or for ex-vivo tissue analysis. It is not possible to directly attach this type of camera to medical apparatus, like laparoscopes or intraoperative microscopes, due to their inability to perform spatial scanning. Some examples of HS acquisition systems, based on push-broom cameras, can be found in Figure 3A,B, while the intraoperative use of these systems are presented in [20,40]. Furthermore, it is possible to use this kind of camera for registering pathological slides [41], as can be observed in Figure 3C.

On the other hand, *spectral scanning* cameras employ an optical element that filters the incoming radiation, registering the entire spatial information of a single wavelength at each and every moment. Capturing an HS cube requires change of the tuned wavelength of the filter in order to perform spectral scanning. There are several types of spectral scanning cameras. The filter wheel cameras require the manual shift of the optical filter, while the Liquid Crystal Tunable Filter (LCTF) or the Acousto-Optic Tunable Filter (AOTF) are devices where the spectral transmission can be electronically controlled [42]. These cameras have lower spectral resolution than the push-broom cameras, and are not suitable for applications where the captured object is moving, because the spatial information may vary for different wavelengths. Nevertheless, these cameras can be easily attached to medical instruments and can offer high spatial resolutions. An example of HS acquisition system for medical applications using these cameras is shown in Figure 3D [43].

The remaining type of HS cameras is called snapshot [44]. Snapshot technology is intended to deal with the main limitation imposed by the previously described HS technologies: real-time acquisition. It is not possible to collect HS or MS data in real-time using the above-mentioned HS technologies for the requirement of performing a scan (either spatial or spectral). These technologies are restricted to static situations, or scenarios where the object that is moving has a slightly lower speed compared to the scan speed. For these reasons, where necessary to obtain HS data of non-static scenes (e.g., living cell imaging) a snapshot camera must be employed. Furthermore, snapshot cameras can be directly attached to clinical instrumentation, such as endoscopes or laparoscopes. Nevertheless, both the spectral and the spatial resolution of the snapshot cameras are lower compared to the other HS technologies. To the best of our knowledge, there is no current research in GI using snapshot cameras, mainly because all preliminary exploration of HS technology in GI is focused to prove the capabilities of the technology for diagnosis, and hence it is necessary to evaluate each scenario using high performance spectral and spatial instrumentation.

## 4. Hyperspectral Image Analysis

As mentioned in the previous sections, HSI data facilitates the identification of different materials. However, to successfully retrieve useful information from HS images, the application of appropriate image analysis techniques is necessary. In this section, a brief overview of such techniques is provided. They include pre-processing algorithms, e.g., for noise removal (HS images carry noise that may affect information extraction) [45,46], HSI system calibration (with respect to the camera spectral range and resolution) [47], feature extraction [48], dimensionality reduction [49], classification [50], spectral unmixing [51], and Normalized Difference Index (NDI) estimation [52,53].

Data acquired using HS instrumentation is highly biased by both the instrumentation and the environmental conditions. In order to remove the influence of instrumentation (mostly the wavelength dependencies of the sensor and grating efficiency and transmission of the lens), is common to perform a calibration. The typical calibration procedure in HS and MS imagery consist of capturing a reference image using a material that has a flat spectral response (e.g., Spectralon). This reference image captures the spectral dependencies of the instrumentation and is used to remove the influence of the instrumentation in the captured HS images.

To ameliorate the challenges imposed by the high dimensionality of HS data, feature extraction and dimensionality reduction approaches are usually employed. Firstly, feature extraction (or band selection) methods are used to select a subset of the original spectral data that contains the most useful information for data exploitation. This reduced set of spectral bands strongly depends on the nature of the specimens under study. Secondly, dimensionality reduction approaches aim to find a representation of HS image data with a lower dimensionality than the original data, while maintaining the most significant information. In HSI, data reduction techniques are widely used for finding new data representations prior to the application of other data analysis techniques (such as classification). This procedure reduces the complexity of the classification task, and it can also contribute to better data visualization or compression [54,55].

One of the key topics in HS image analysis is classification, which aims for the identification of the materials depicted within an HS image. HS data classification methods can be categorized into supervised and unsupervised. Supervised classifiers require training using prior information on the materials to be classified; hence, a mathematical model is optimized using this information. Then, this model is able to infer predictions about new data. A recent review article by Ghamisi et al. [56] analyzes the mostly extended supervised classifiers employed by the HS community. Unsupervised classification methods (also known as clustering methods [57]) have the goal of grouping pixels according to some spectral similarity criteria. Although these kinds of algorithms provide useful information about the materials that are present in a scene, it is not possible to relate these groups of similar pixels with their class membership. Recent studies have shown that the joint exploitation of the spectral and the spatial information in HS images improves the classification performance [58].

Finally, spectral unmixing and NDI estimation have been used for HS image analysis. On the one hand, spectral unmixing techniques, such as those based on Linear Mixture Models (LMMs), make the assumption that each pixel of an HS image can be modeled as the weighted sum of pure spectra elements (called endmembers). This technique tries to overcome the limited spatial resolution that generally characterizes MS and HS imaging compared to the traditional RGB imaging. Unmixing algorithms first find the endmembers and then estimate the abundance (proportion) of each endmember in a single pixel [51]. On the other hand, NDI-based approaches try to establish a combination of spectral channels that reveal some characteristics of the subject under study. For example, the Normalized Difference Vegetation Index (NDVI) aims to assess the presence of live vegetation in HS satellite images [52]. In the context of medical applications, a Melanoma Identification Index has been proposed in [53] for identification of skin lesions in dermoscopic HS images. Additionally, there are some researches that make use of Light Transport Models in tissue to retrieve useful information about tissue diagnosis [16,17,59,60].

## 5. Hyperspectral Imaging in GI Diagnosis

**Figure 4:**
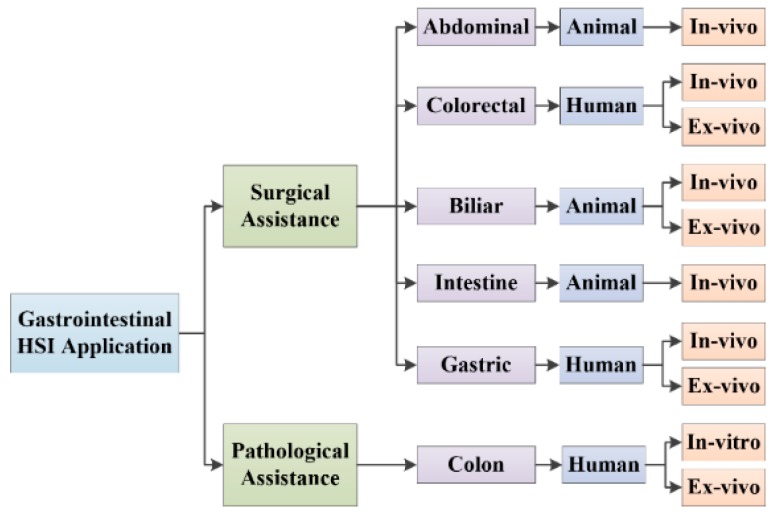
Taxonomy of the current gastrointestinal HSI applications.

HSI is an emerging technology still at an early application stage in the medical field. Therefore, the number of publications regarding the use of this technology in gastroenterology is limited. This section summarizes the main research works performed in this field, structured following the taxonomy presented in Figure 4. This taxonomy divides the gastrointestinal HSI applications categorized by the type of application, the type of subject to study and the type of sample (i.e., the organ where this technology is applied).

### 5.1. Surgical Assistance in Real-Time

One of the current target applications of medical HSI is in the field of surgical guidance. Such applications are motivated by the non-invasive nature of HSI technology, and for its capability of generating an alternative visualization of tissues, that can assist in the identification of several GI diseases. In this section, we present the most important surgical guidance tools based on HSI developed for GI use.

#### 5.1.1. Abdominal Organs Differentiation

**Figure 5:**
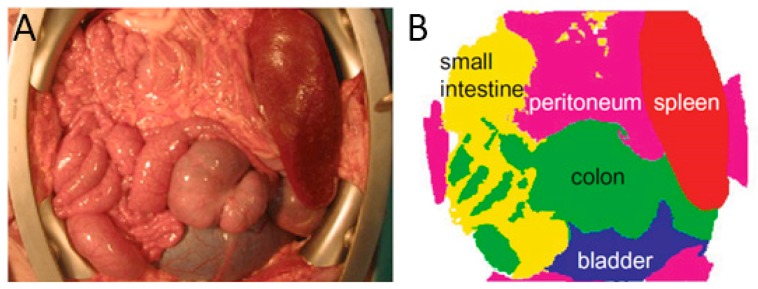
Use of HSI for organs identification in pig’s abdominal surgery performed in [61]. (**A**) RGB image from pig’s abdominal cavity and organs; (**B**) Segmentation map obtained after HS image processing and analysis, where the different organs are identified using different colors.

An illustrative use of HSI as a visual guide tool during surgery can be found in [61]. In this research work, the authors collected and processed spectral signatures from various abdominal organs. The experiment was performed during an open abdominal surgery on a pig. By processing the spectral signatures of the small intestine, colon, peritoneum, bladder and spleen, a thematic map where each organ is identified was generated. The results of this thematic map can be found in Figure 5, where each organ is represented with a different color. The automatic identification of different tissues during surgery may extend the surgeon’s visual capabilities, making possible examining larger areas of tissue, and therefore saving surgical time.

#### 5.1.2. Colorectal Surgery

**Figure 6:**
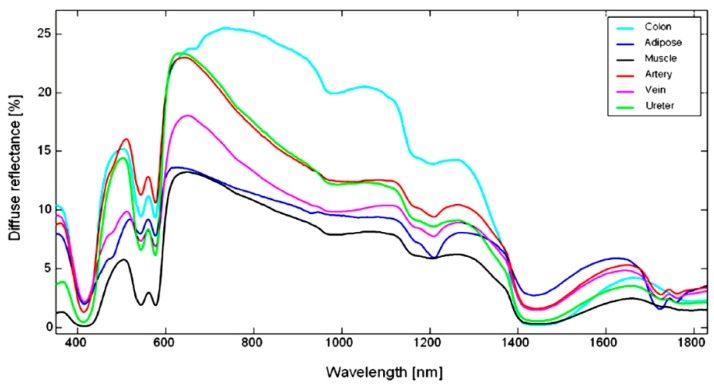
Mean spectra per tissue type acquired during colorectal surgery in [62]. Average tissue spectra for ureter (green), mesenteric adipose tissue (dark blue), artery (red), colon (light blue), muscle (black), and vein (purple).

Colorectal surgeries have also been studied using HSI as a guidance tool during tissue resections. Schols et al. [62] presented an explorative study aiming to collect and automatically differentiate five different tissue types within the human abdomen: colon, muscle, artery, vein and mesenteric adipose tissue (Figure 6). This tool could help surgeons to avoid ureteral injuries, which may lead to severe complications such as intra-abdominal sepsis, renal failure or loss of renal functions. Near-infrared (NIR) fluorescence imaging has been also used to enhance the visualization of the ureters and arteries. However, this technique requires the use of a contrast agent. HS images from 10 human patients were collected and analyzed to verify whether HS images were a suitable tool for identifying arteries and ureters intraoperatively. Although the spectral signatures collected from various organs presented similarities, the authors reached promising results in the automatic discrimination between different tissues. Therefore, the foundations for a non-invasive optical guidance tool that could be used during colorectal surgery, enhancing the visualization of critical anatomy, have been laid. The same research group also studied an approach to automatically identify different tissue types that can be observed during laparoscopic colorectal surgery procedures [63]. Five types of tissue were recorded from ex-vivo human resected specimens, i.e., mesenteric fat, blood vessels, ureter, colonic tissue and tumorous colonic tissue. The data acquisition was carried out by using a spectrometer working in the spectral range 440–1830 nm. Based on the measured spectral signatures, the authors posed that the differentiation between tissues is possible by exploiting the spectral fingerprints of each tissue.

The above-mentioned research consisted on analyzing the capabilities of HSI to differentiate between different types of tissue. Nevertheless, this technology has been also used for colon diagnosis applications. Malignant colorectal tumors, adenomatous polyps and different types of colorectal normal mucosa were analyzed in [64]. To collect in-vivo HS data, a contactless endoscopic diagnosis support system was attached to an HS camera. The endoscopic system was able to capture HS images in the wavelength range from 405 to 665 nm, acquiring 27 spectral bands using a filter wheel. A total of 21 HS cubes from 12 different patients were employed to assess an innovative band selection algorithm based on Recursive Divergence Method (RDFS). In order to evaluate the performance of the proposed algorithm, a supervised classification method based on a Support Vector Machine (SVM) classifier was used, with the manually labeled spectral samples of the different types of tissue acquired, to serve as a training set. Using only five bands from the original 27, the HSI system was able to identify in real-time the colorectal tumors and outlining the region affected by the tumor with an average accuracy of 92.9 ± 5.4%. Furthermore, only these five bands were sufficient for the enhancement of the visualization of the microvascular network on the mucosa surface. Another approach for colonic cancer detection using HSI can be found in [65], where the authors study the identification of esophageal squamous neoplasm by using an HS endoscopic imaging system.

Finally, although the work performed by Beaulieu et al. [66] cannot be considered strictly as HSI (they use a spectrometer, so there is no spatial information), they present an interesting discussion about which spectral range provides a better discrimination between tumor and normal colon tissues. After the analysis, they concluded that the inclusion of SWIR (Short-Wave InfraRed) spectral bands contribute to a better discrimination of malignant and normal tissue.

#### 5.1.3. Bowel Anastomosis

**Figure 7:**
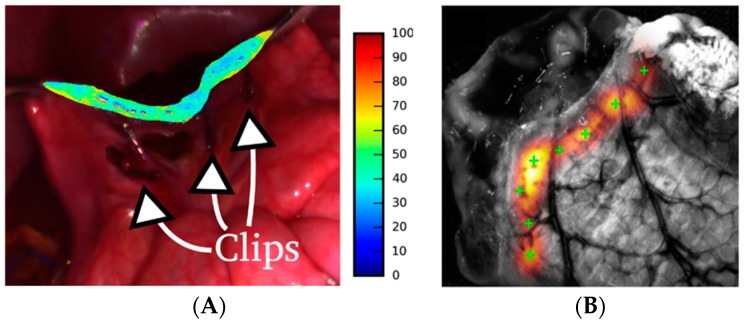
HSI application in Bowel anastomosis. (**A**) Small bowel oxygenation estimation shown in [68]; (**B**) Suture map and recommendations about the location of the suture provided in [69].

The correct monitorization of oxygenation and blood volume fractions are key for success in colorectal surgery. For this reason, some researchers have focused their attention on imaging visualization tools that can prevent surgical complications such as intestinal anastomosis. In [67,68] the authors proposed methods to derive both the oxygenation of tissue and the blood volume fraction by using models for light transportation in tissue. The results are shown to surgeons as thematic maps where these physiological parameters are presented (Figure 7A). The work presented in [69] go beyond, and propose a method to suggest the optimal location of sutures for a better surgical outcome. To generate such map (Figure 7B), the authors made use of the information about the blood-vessels location and the tissue thickness (measured from a MS image). Although these research works have been only tested in swine models, they show a promising methodology for improving colorectal surgeries. Another interesting application of MSI in bowel anastomosis can be found on [70]. In this research, authors noticed that the variations in the measured reflectance spectra using an MSI laparoscope are coherent with biophysical changes during small bowel radiofrequency fusions.

#### 5.1.4. Biliary Anatomy Identification

Currently, the technologies used to delineate the anatomy of the biliary tree, such as the Intraoperative Cholangiography (IOC) or the routine intraoperative ultrasonography, are not sufficiently accurate. For this reason, the biliary anatomy has been also investigated using HSI to accurately identify the different parts of the anatomic structure. Concretely, in [71] and [55], Zuzak et al. proposed to use HSI to develop a visualization system capable of identifying the biliary trees that do not depend on any prior dissection. The authors developed an acquisition system consisting of an HS camera coupled with a conventional laparoscope that was intended to enhance the visualization of the biliary anatomy. The spectral range of this device covers the EM spectrum from 650 to 1100 nm. This technology may achieve a reduction of bile duct injuries during surgery. In order to test the ability of HSI in detecting the bile tree, a study visualizing intraoperatively the abdominal organs of pigs during close cholecystectomy procedures was carried out. The authors found that the measured spectra of several anatomic structures are unique, allowing the differentiation of arterial vessels, venous structures and bile duct. In [55], the HS images were processed using PCA (Principal Component Analysis), which proves a visual enhancement of the different observed anatomical structures, i.e., the gall bladder and the liver. The correct identification of the tissue types was assessed by using the morphological structures of each tissue. This research work shows that the visualization of the biliary tree could be safely performed during surgical procedures without the need for prior imaging. The inclusion of this technology may lead to eliminating the risk of the bile duct injury during cholecystectomy, avoiding also the current need of injecting radioactive contrast agents.

In addition, dual-mode imaging systems have been using for this goal. Mitra et al. [72] used a system composed by a Indocyanine-green-loaded (IGC) micro-balloon and an HS camera. The goal of this study was to identify the surrounding anatomy during IOC in real-time. This imaging technique was tested over ex-vivo swine tissues, showing an accurate identification of the biliary anatomy. The advantages of this imaging modality over IOC are its low cost, its real-time response, and its independence of radiation agents. This type of visualization tool can be used for guidance during surgical procedures. Furthermore, it can be used as an additional input to surgical robots, such as the Da Vinci robot [73].

#### 5.1.5. Intestinal Ischemia Identification

The intestinal ischemia can be defined as an inadequate blood flow to the intestine, causing an inability to absorb food and nutrients, bloody diarrheal, infection and gangrene. In this sense, HS technology has been also used for this application. Akbari et al. [74] developed a intraoperative HSI tool capable of obtaining spectral signatures of intestinal ischemia acquired during a pig abdominal surgery. Two cameras were used, covering from 400 to 1700 nm. The methodology followed in this paper to process the HSI data consisted in finding an optimal NDI that allows the discrimination of intestinal ischemia over other kind of tissue. This article demonstrated that the HS image analysis is suitable for visualizing intestinal ischemia during surgical procedures. However, although this study was performed with a wide spectral range and presents good analysis of the capabilities of HSI technology, the instrumentation based on push-broom cameras is extremely outsized, being inappropriate for clinical environments.

#### 5.1.6. Gastric Cancer Identification

Other relevant research where HSI is used to study GI diseases can be found in [75], where the authors describe the use of HSI for detecting human gastric cancer. This study was carried out over ten patients who underwent a total gastrectomy. The HS images were captured ex-vivo after the resection of a tumor using an HS camera covering the range from 1000 to 2500 nm. After pathologic diagnosis, the real diagnosis was compared with the image processing results. Although the data acquisition system was not appropriate to be used for endoscopic diagnosis (it consisted on a proof-of-concept demonstrator), the data analysis enabled the identification of wavelengths improving the differentiation between healthy and tumor tissues. These wavelengths can be employed as specifications in the development of future laparoscopic HSI systems optimized for gastric cancer detection.

Another application of HSI as a guidance tool during surgical procedures can be found in [76]. In this research work, the authors processed the HS data aiming to determine a combination of wavelengths, highlighting the presence of ulcer regions in gastric tissue. Using different spectral components from different types of tissue, the authors generated a thematic map where the visualization of ulcer and erythematous regions of the image were able to be differentiated with respect to the surrounding tissue. Other research works have also studied the in-vivo identification of gastric ulcers using HSI [77] or gastric cancer employing a customized MSI video endoscopy system capable of capturing multispectral video composed by six bands located in the visual spectral range [78]. These research works suggest that HSI and MSI can be used as a guidance tool both for diagnosing, and for delimitating the gastric tumor margins accurately.

### 5.2. Pathological Assistance

The research works previously presented investigate methods aiming to automatic identification and visualization of different types of tissues, in the context of clinical diagnosis, mainly to facilitate surgical procedures in real-time. Nevertheless, HS images have been also applied to the pathological diagnosis of colonic diseases. The following papers describe the use of HSI for the identification of tumorous tissues from in-vitro and ex-vivo human colon pathology samples.

The work described in [79] presents a study performed on biopsy slides of colon tissue aiming to distinguish between normal and malignant cells through exploiting HSI. A morphological analysis of the HS images of biopsy slides with several microdots belonging to different patients was performed employing a dimensionality reduction and cellular segmentation to describe the shape, orientation and other geometrical attributes, using ICA (Independent Component Analysis) and the k-Means algorithm. The segmentation maps obtained after the application of k-Means clustering algorithm allow differentiating the malignant and benign cells by their morphological features. These morphological features were then used as input to a classification process that differentiates between normal and malignant cells. For this purpose, LDA (Linear Discriminant Analysis) algorithm was used due to its reduced computational cost and acceptable performance. The images were captured with an HSI system based on a tunable light source and a CCD (Charge-Coupled Device) camera coupled to a microscope with a magnification of 400×, covering the spectral range between 450 and 850 nm. An accuracy of up to 84% was obtained in the classification experiments, demonstrating that the use of HSI facilitates the discrimination between normal and malignant cells of colon tissue by using its morphological features.

Furthermore, Rajpoot et al. [80] employed an SVM to classify in-vitro samples of normal and malignant human colon cells. Archival Hematoxylin and Eosin (H&E) stained micro-array tissue sections of normal and malignant (adenocarcinoma) colonic tissue image data cubes were acquired at microscopic level. The spatial dimensions of each HS cube were 1024 × 1024 pixels, having 20 spectral bands covering the wavelength range from 450 to 640 nm. Multiscale morphological features such as area, eccentricity, equivalent diameter, Euler number, extent, orientation, solidity, major axis length, and minor axis length, were obtained from the segmented maps to be used in the SVM classification procedure. The experiments carried out in this study reveal that an accurate discrimination (99.72%) between normal and malignant tissue can be achieved.

Colon biopsy samples have also been studied by Masood et al. [81], where the authors propose an algorithm for the automatic classification of colon biopsies based on spatial analysis of HS images captured from colon biopsy samples. The aim of their work was to distinguish between benign and malignant tissue. Although the authors collected HS cubes with 128 bands, they only use a single spectral band. To this end, the processing framework consisted on selecting a single band and performing a spatial analysis of this image by using Circular Local Binary Patterns (CLBPs). Then this information is used by different supervised classifiers in order to retrieve diagnostic information from colon biopsies. The maximum accuracy obtained was 90.6% for the SVM classification, with 87.5% sensitivity and 93.7% specificity. A clear advantage of performing the spatial analysis on a single band is to save acquisition, storage, and computational costs, but it is difficult to state that this algorithm really makes use of the richness of information contained in the HS images. Although authors reached good discrimination between malignant and benign tissue, the discrimination capabilities may increase if more spectral channels are used.

HSI technology was also evaluated for differentiating normal and cancerous gastric cells in H&E stained pathological slides [82]. In this case, the main motivation to perform the study was to analyze the differences in pH levels between cancerous and normal cells. These pH differences were reflected in the spectral signature of the different cells, providing good discrimination between malignant and normal cells using only the spectral information of cell nuclei. Conversely, the work presented by Hidovic-Rowe et al. [59,60] aimed to extract histological parameters from ex-vivo colon tissues using HSI. The measured parameters were the blood volume fractions, the hemoglobin saturation levels and the size of collagen fibers or the thickness of the mucosa layer. These parameters were computationally estimated by using a light transportation model over colon tissue, identifying both normal and tumor tissues.

**Figure 8:**
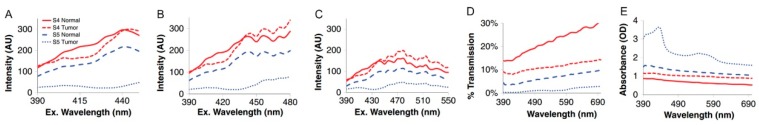
Spectral differences between normal and cancerous tissue for two specimen pairs: S4 and S5 presented in [83]. Preliminary data demonstrate the differences in spectral signature between cancerous and normal tissue. Transmission and absorbance spectral data indicate that adenocarcinoma displays increased optical absorbance, as compared to surrounding normal tissue, with additional spectral differences that could be exploited to increase sensitivity and specificity for tumor detection. Spectral scan types are as follows: (**A**) Fluorescence excitation scan from 390 to 450 nm; (**B**) Fluorescence excitation scan from 390 to 480 nm; (**C**) Fluorescence excitation scan from 390 to 550 nm; (**D**) Transmission scan from 390 to 700 nm; (**E**) Absorbance scan from 390 to 700 nm.

Some current technologies used to improve colorectal exams are the White Light Endoscopy (WLE), the Chomoendoscopy, Autofluorescence Imaging and Narrow Band Imaging. Nevertheless, these technologies present limitations that motivate the finding of new technologies to this end. Motivated by this fact, the work presented in [83] evaluated the initial feasibility of using HSI for colonic adenocarcinoma identification using HSI fluorescence excitation-scanning for measuring changes in fluorescence excitation spectrum. To this end, an ex-vivo and in-vitro analysis of colonic tissue was carried out, in order to determinate if HSI fluorescence can be used as an additional endoscopy technology. A total number of eight patients were enrolled in that study. Specimens were imaged using a custom HSI fluorescence excitation-scanning microscope system. As it can be seen in Figure 8A, at short excitation wavelengths, the fluorescence total intensity of adenocarcinomas was lower than normal tissue. However, fluorescence resulting from excitation at higher wavelengths was increased, and in the S4 sample was higher than normal tissue, Figure 8B,C. Transmission and absorbance spectral data indicate that adenocarcinoma displayed increased optical absorbance, as compared to surrounding normal tissue (Figure 8D,E). These preliminary data suggest that there are significant differences in the spectral signature of cancerous and normal tissue. In this sense, the same research group continued the investigations and presented new results in [84,85]. These results could pave the way towards advanced classification systems than can automatically identify tissues attending to their spectral signatures.

In addition to the studies presented before, there are other interesting and novel applications of pathological assistance using MSI/HSI. For example, authors in [86] collected MS images from four types of colorectal cells: viz. normal, hyperplastic polyps, tabular adenoma with low grade dysplasia and carcinoma. After MSI analysis of different colorectal tissues, an accurate discrimination using colorectal cells was achieved.

**Figure 9:**
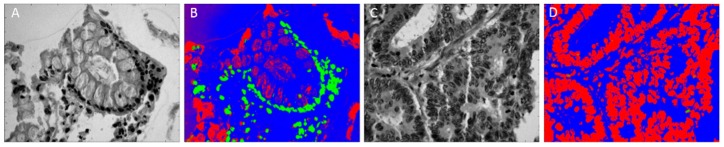
Samples of HS normal and abnormal colonic tissue images and the results obtained after applying the pre-processing sonification presented in [87]. (**A**) Sample specimen showing normal colonic tissue in gray-scale image; (**B**) Final three-dimension image that gives the probabilities that the point in the specimen belongs to normal colon tissue; (**C**) Sample specimen showing abnormal colonic tissue in gray-scale image; (**D**) Final three-dimension image that gives the probabilities that the point in the specimen belongs to abnormal colon tissue.

Finally, another less conventional approach, investigated in the context of HSI for GI pathological assistance, presents a vocal synthesis model and its application to sonification of HS colonic tissue images [87,88]. The authors state that sonification could be used as an intuitive means of representing and analyzing high-dimensional and complex data. The high-dimensional data for sonification have been obtained from HS scans of normal and abnormal colon tissue, the abnormal tissue being potentially cancerous. The tissue images were collected in cooperation with the Department of Applied Mathematics at Yale University. A series of slides were prepared from distinct patients, containing more than 300 microdots each slide, and each microdot corresponding to a slice of colon tissue (roughly 0.5 × 0.5 mm in size). Each microdot may contain either normal or malignant colon tissue. A slide was chosen and illuminated with a tuned light source (capable of emitting any combination of light frequencies in the range of 450 to 850 nm), and the transmitted image was magnified 400× by a Nikon Biophot microscope. An amount of 15 data cubes of normal colonic tissue and 46 data cubes of abnormal colonic tissue were collected. Examples of pre-processed specimens are illustrated in Figure 9. Initial experiments with a variety of vocal tract models suggest that human ability to easily identify vowel-like sounds is promising for intuitive sonification.

### 5.3. HSI Application Summary

Finally, in Table 1 we provide a summary of the most relevant research work in the field of gastroenterology using HSI. This table is organized attending to: (1) the disease that has been investigated; (2) the type of tissue involved in each study; (3) the subject of the research; (4) the HSI technology employed to acquire the images; and (5) the data processing methods applied to extract useful information from HS data.

## 6. Discussion

Although HSI technology has shown its potential to be used as a diagnostic tool, the roadmap for a new generation of HS medical devices is not clear yet. One of the most relevant challenges holds in the acquisition system. The most appropriate technology to be used in the GI tract is not clear. Although most of the state-of-art studies employ systems based on LCTF, acquisition systems based on push-broom or tunable light sources have also been successfully employed to this end. Despite the fact that higher spectral resolution is achieved using push-broom cameras, the spatial scanning required to obtain an HS cube makes difficult the integration of such kind of cameras with standard medical instrumentation, such as gastroscopes, colonoscopes, and laparoscopes. For this reason, LCTF can be regarded as the most extended technology for the GI tract. On the other hand, a novel study has been recently performed to identify in-vivo esophageal squamous neoplasia in human patients by using a RGB-HSI combined system [65]. In this study, authors developed a system capable of artificially generating an in-vivo HS image in the visual range by merging the information of a RGB endoscopic image with the spectral information obtained from a 30 Macbeth color checker tile measured with a spectrometer.

Concerning the optimal spectral range for GI diagnosis, the answer is still unclear. Although most of the studies in the state-of-art use images in the VNIR spectral range, there are several research works reporting successful detection of diseases using a spectral range beyond 1000 nm. In the upcoming years, the research community should assess the optimal spectral range for diagnostic applications. Depending on this spectral range, the directions on medical HS acquisition systems will possibly vary. On the one hand, if the VNIR spectral range is optimal for disease detection in the GI tract, the future medical HS images will be probably based on LCTF or snapshot cameras, that provide lower spectral resolution and a limited spectral range compared with push-broom cameras, but they are easily adapted to conventional medical instrumentation. On the other hand, if the spectral range increases beyond 1000 nm, the identification of diseases will probably improve due to the richer amount of available spectral information. However, push-broom cameras have to be used, and the engineering challenge will be the adaptation of the push-broom cameras to conventional medical instrumentation.

Recently, pioneering HSI-enabled flexible endoscopes and concept capsule endoscopes have been proposed [47,91,92], indicating the feasibility of incorporating HSI in clinical practice for colorectal cancer detection. Future challenges of HSI in gastrointestinal endoscopy are mainly associated with its application for the detection and characterization of various different kinds of abnormalities.

There are other interesting applications of MSI/HSI that are closely related to gastroenterology, but they are out of the scope of this manuscript. For example, in a recent study performed by Bhutiani et al. [93], the authors studied the in-vivo detection of AF-680 dye encapsulated PLA (Polylactic Acid) using an MSI laparoscope. The exploitation of such type of information has a potential use for in-vivo characterization of drug delivery.

Besides, on a recent review regarding current trends in endoscopic imaging, Joshi et al. [94] mentioned two novel applications of MSI that are relevant to be mentioned in this review. The first application is related to the use of dual-channel fluorescence images from in-vivo cross sections using a confocal microendoscope [95]. This research points out that in-vivo cross section images can be captured with a similar orientation as the corresponding histological sample. The same methodology could be applied with MSI/HSI instead of fluorescence. The second application presented by Joshi et al. [96] used a multispectral endoscope to simultaneously collect three fluorescence images (DEAC, 6-TAMRA and CF633). They were able to acquire images from colonic adenoma stained with two different peptides at the same time, providing sharp visualization of the lesion margins.

Structured light and MSI were used to simultaneously extract information about reflectance and surface structure of tissue during small bowel surgery [97]. Although this research was just a proof-of-concept, the incorporation of an additional spatial dimension to MSI/HSI can lead to better discrimination among different tissues.

Other spectral technologies based on Raman spectroscopy and Quantum Cascade Lasers (QCL) have been applied to assist pathological analysis. First, Raman spectroscopy has been employed to detect alterations in the biomedical composition of intestinal tissue biopsies, which can reveal coeliac disease [98]. Although more research is needed to confirm the hypothesis, Raman spectroscopy is also presented as a promising alternative for coeliac disease detection. Furthermore, several research works have been performed in the literature with the goal of diagnosing histological slides without requiring stains using QCL acquisition systems. These systems are able to acquire hyperspectral images beyond five microns. Firstly, in [99], Kröger-Lui et al. had the goal of detecting goblet cell regions in colonic epithelium using unstained histological sections, instead of conventional H&E stained samples. They demonstrated a strong correlation between the contents obtained with HS unstained images and the corresponding stained section using H&E. Secondly, in [100], Petersen et al. presented a proof-of-concept following a similar methodology, demonstrating that the mid-infrared information could be also useful for diagnosis purposes. In this pilot study, the authors found valuable information about protein rich amide regions of colonic crypts, the musin secretions and the surface epithelium walls that could be extracted from unstained colon sections using this spectral range (beyond 2500 nm).

As far as the data analysis techniques are concerned, there is not a generalized framework for processing HS data. Table 1 summarizes the data analysis methods currently used in GI HS applications. Actually, most techniques aim to get an enhanced visualization of tissues, with dimensional reduction techniques, such as PCA, LDA or ICA, being the most popular HS data processing methods. Another interesting trend is the definition of some normalized difference indexes to retrieve some characteristics of tissues, such as the proposed Normalized Difference Ischemia Index (NDII) or the Normalized Difference Cancer Index (NDCI). Although the use of such types of indices is handy, their use is still limited. As far as classification methods are concerned, in contrast to other HSI applications (such as precision agriculture or food quality analysis) the classification approaches used in the context of GI endoscopy imaging are limited, mainly based on SVMs. Maybe the slow raise of HS data classifiers for GI HS data is motivated by the difficulties to collect sufficiently large labeled datasets, allowing the generation and evaluation of reliable classification models. A relevant challenge for the analysis of medical HS data would be to investigate adaptation or enhancements of the current state-of-art HS data classification approaches (mainly coming from the Remote Sensing community) for the analysis of GI endoscopy data.

## 7. Conclusions

This survey is intended to provide a useful introduction to HSI in the medical field, paying special attention to the applications in gastroenterology. HSI has been limitedly explored for clinical purposes in GI endoscopy; moreover, the study of the literature indicates that it is a novel imaging modality with a high potential to improve several current medical procedures. For instance, HSI can contribute to make gastric surgical procedures safer by avoiding bile duct injury or ureteral injuries. Furthermore, it can contribute in a more accurate determination of tumor boundaries, facilitating a complete resection of the tumor tissue. Detection of malignant tissue, beyond the limitations of the contemporary white light imaging remains an area where properly employed HSI could lead to precision diagnosis. Further use of HSI technology has to face limitations of space and applicability [101].

## Figures and Tables

**Table 1:** Summary of HSI applications in gastroenterology.

Application/Disease	Spectral Range (nm)	HSI Technology	Experiment Type	Study Subjects	Data Analysis Methods (Category */Method ^¥^)	Reference(s)
Biliary tree visualization	650–1050	LCTF	In-vivo	Swine	D, E/PCA	[55]
Colon cancer detection	400–700	LCTF	Ex-vivo	Humans	F, E/LPM	[59,60]
Organs identification during surgery	900–1700	Push-broom	In-vivo	Swine	SA, P/DWT; C/SOM	[61]
Identifying tissues during surgery	350–1830	DRS	In-vivo	Humans	SA, F/SGAD; C/SVM	[62]
Tissue identification during colorectal surgery	440–1830	DRS	Ex-vivo	Humans	SA, C/TPCR	[63]
Malignant colorectal tumors and adenomatous polyps	405–665	Filter Wheel	In-vivo	Humans	R/RDFS; C/SVM	[64]
Colon cancer detection	300–1800	Spectroscopy	Ex-vivo	Humans	C/LDA; C/SVM	[66]
Oxygenation measurement (small bowel)	400–720	LCTF	In-vivo	Swine	Ex/Linear light model	[67]
Oxygenation measurement (small bowel)	470–700	Filter-based	In-vivo	Swine	Ex/Non-linear light model	[68]
Suture recommendation (intestinal anastomosis)	470–770	LED-based	Ex-vivo	Swine	Ex/2D-filtering, SAM and composite images from the multispectral image	[69]
Monitoring radiofrequency fusions in small bowel	460–700	LCTF	In-vivo	Swine	Ex/Linear light model	[70]
Biliary trees identification	650–1100	LCTF	In-vivo	Swine	D, E/PCA	[71]
Biliary anatomy visualization	650–700	LCTF	Ex-vivo	Swine	S/LMM, R/PCA	[72]
Intestinal ischemia identification	400–1700	Push-broom	In-vivo	Swine	I/Ischemia Index; C/SVM	[74]
Gastric cancer detection	1000–2500	Push-broom	Ex-vivo	Humans	I/Cancer Index; C/SVM	[75]
Gastric ulcers	405–665	Filter Wheel	In-vivo	Humans	R, E/DI	[76]
Gastric cancer	400–800	N/A	Ex-vivo	Humans	C/MDC	[77,89]
Gastric cancer	400–650	Tunable Light Source	In-vivo	Humans	C/SVM; C/RF; C/RobustBoost; C/AdaBoost	[78]
Colon cancer detection	450–850	Tunable Light Source	In-vitro	Humans	R/ICA; R/PCA; C/*k*-Means; C/LDA; C/SVM	[79,80]
Colon cancer detection	440–700	Tunable Light Source	In-vitro	Humans	F/CLBP; R/PCA; C/LDA; C/SVM	[81]
Gastric cancer cell identification	420–720	LCTF	In-vitro	Humans	R/Manual band selection; C/ANNs	[82]
Colonic adenocarcinoma identification	390–700	LCTF	Ex-vivo	Humans	SA	[83]
Colon cancer detection	360–550	LCTF	In-vitro	Humans	S/LMM; R/PCA	[84,85]
Colorectal cell differentiation	400–1700	LCTF	In-vitro	Humans	F/LBP, C/RF	[86]
Colon cancer detection	400–1000	Push-broom	Ex-vivo	Humans	DR/SPA; C/LDA	[90]

* Categories of data analysis methods: (P) Preprocessing; (F) Feature extraction; (C) Classification; (R) Data Reduction; (S) Spectral Unmixing; (I) Normalized Difference Index; (E) Tissue Visualization Enhancement; (SA) Spectral Signature Analysis; (Ex) Exploratory Data Analysis. ^¥^ Data analysis methods: (SGAD) Spectral Gradients and Amplitude Differences; (SVM) Support Vector Machines; (DWT) Discrete Wavelet Transformation; (SOM) Self-Organizing Maps; (CLBP) Circular Local Binary Patterns; (PCA) Principal Component Analysis; (LDA) Linear Discriminant Analysis; (LPM) Light Propagation Modeling; (LMM) Linear Mixture Model; (ICA) Independent Component Analysis; (RDFS) Recursive Divergence Feature Selection; (DI) Dependence of Information; (MDC) Minimum Distance Classifiers; (TPCR) Total Principal Component Regression; (RF) Random Forest; (SAM) Spectral Angle Mapper; (SPA) Successive Projection Algorithm; (LBP) Local Binary Pattern; (ANNs) Artificial Neural Networks.
